# Altered levels of serum sphingomyelin and ceramide containing distinct acyl chains in young obese adults

**DOI:** 10.1038/nutd.2014.38

**Published:** 2014-10-20

**Authors:** H Hanamatsu, S Ohnishi, S Sakai, K Yuyama, S Mitsutake, H Takeda, S Hashino, Y Igarashi

**Affiliations:** 1 Laboratory of Biomembrane and Biofunctional Chemistry, Graduate School of Advanced Life Science and Frontier Research Center for Post-Genome Science and Technology, Hokkaido University, Sapporo, Japan; 2 Department of Gastroenterology and Hepatology, Hokkaido University Graduate School of Medicine, Sapporo, Japan; 3 Health Care Center, Hokkaido University, Sapporo, Japan; 4 Department of Applied Biochemistry and Food Science, Faculty of Agriculture, Saga University, Saga, Japan; 5 Laboratory of Pathophysiology and Therapeutics, Faculty of Pharmaceutical Sciences, Hokkaido University, Sapporo, Japan

## Abstract

**Objective:**

Recent studies indicate that sphingolipids, sphingomyelin (SM) and ceramide (Cer) are associated with the development of metabolic syndrome. However, detailed profiles of serum sphingolipids in the pathogenesis of this syndrome are lacking. Here we have investigated the relationship between the molecular species of sphingolipids in serum and the clinical features of metabolic syndrome, such as obesity, insulin resistance, fatty liver disease and atherogenic dyslipidemia.

**Subjects:**

We collected serum from obese (body mass index, BMI⩾35, *n*=12) and control (BMI=20−22, *n*=11) volunteers (18−27 years old), measured the levels of molecular species of SM and Cer in the serum by liquid chromatography-mass spectrometry and analyzed the parameters for insulin resistance, liver function and lipid metabolism by biochemical blood test.

**Results:**

The SM C18:0 and C24:0 levels were higher, and the C20:0 and C22:0 levels tended to be higher in the obese group than in the control group. SM C18:0, C20:0, C22:0 and C24:0 significantly correlated with the parameters for obesity, insulin resistance, liver function and lipid metabolism, respectively. In addition, some Cer species tended to correlate with these parameters. However, SM species containing unsaturated acyl chains and most of the Cer species were not associated with these parameters.

**Conclusions:**

The present results demonstrate that the high levels of serum SM species with distinct saturated acyl chains (C18:0, C20:0, C22:0 and C24:0) closely correlate with the parameters of obesity, insulin resistance, liver function and lipid metabolism, suggesting that these SM species are associated with the development of metabolic syndrome and serve as novel biomarkers of metabolic syndrome and its associated diseases.

## Introduction

Obesity is one of the leading causes of morbidity and mortality, and its prevalence is increasing in the younger population.^1,2^ Because obesity is associated with cardiovascular disease, type 2 diabetes, nonalcoholic fatty liver disease (NAFLD), hypertension and several types of cancer including cancers of the colon, kidney and pancreas, early prevention of obesity has become of great importance.^3,4^ Thus, to identify the factors for the pathogenesis of obesity and to develop methods for diagnosing obesity and its associated diseases at an early stage for their prevention are urgent issues.

Sphingolipids are a class of lipids composed of a backbone of sphingoid bases that are modified to produce ceramide (Cer) and more complex compounds, such as sphingomyelin (SM) and glycosphingolipids.^5^ Sphingolipids have many biological functions, such as membrane structure stabilization, cell-to-cell recognition and signaling.^6,7^ Accordingly, the disturbance of the sphingolipid metabolism contributes to the pathogenesis of several diseases such as Alzheimer's disease and specific types of cancer, as well as sphingolipidoses including Gaucher disease, Niemann–Pick disease and Farby disease.^3,8^ Recently, various studies have revealed that an altered metabolism of sphingolipids is involved in the development of metabolic syndrome. For example, it has been reported that the administration of myliocin, a potent inhibitor of the *de novo* synthesis of global sphingolipids, into rodent models of obesity improves insulin resistance, glucose homeostasis and liver steatosis.^9,10^ We also demonstrated that the synthesis of SM with SM synthase 2 and the phosphorylation of Cer with Cer kinase are related to high-fat diet-induced obesity, fatty liver and insulin resistance in mice.^11,12^

SM, a phosphocholine head group bound to Cer, is associated with lipoprotein particles and is the major sphingolipid in the peripheral blood. The increase in serum SM level has been shown to be a risk factor for cardiovascular diseases.^13,14^ In addition to SM, Cer is also associated with lipoprotein particles and circulates in peripheral blood. Plasma Cer level correlates with the levels of total cholesterol, neutral lipids and fatty acids, and the increase in Cer level could be a risk factor for atherosclerosis.^15^ Several studies of humans and rodents indicated that the increase in plasma Cer level is involved in the development of insulin resistance.^16^ Moreover, Kurek *et al.*^10^ indicated that the Cer level in liver tissue is high in a rat NAFLD model. However, detailed profiles of sphingolipid species including acyl chain length in obesity-related diseases such as insulin resistance, NAFLD and atherogenic dyslipidemia are largely unknown. In this study, we measured the levels of SM and Cer molecular species by liquid chromatography-mass spectrometry (LC-MS) of serum obtained from young obese adults with body mass indices (BMIs) ⩾35 and the control group with BMIs of 20−22 kg m^−^^2^. We then carried out an analysis of the correlation between these sphingolipids and the parameters of insulin resistance, liver function and lipid metabolism. Our results suggest that the high levels of serum SM species containing saturated acyl chains are specifically associated with the development of obesity, insulin resistance, NAFLD and atherogenic dyslipidemia.

## Materials and methods

### Subjects

Twelve students with BMIs ⩾35 kg m^−^^2^ (obese group) were recruited from among the participants of the 2013 annual health check-up held at Hokkaido University, Sapporo, Japan. In addition, 11 age-matched students with BMIs of 20−22 kg m^−^^2^ (control group) were recruited. After the purpose of the study had been explained to all the subjects, written informed consent was obtained. None of the subjects were on any medications or were smokers, and all were free of chronic viral hepatitis and other chronic liver diseases. Their alcohol intake was <140 g per week. This study was approved by the ethics committee of Hokkaido University Graduate School of Medicine.

### Ultrasonography

Ultrasonography was performed in all the subjects in the supine position (LOGIQ S7, GE Healthcare, Little Chalfont, UK). Fatty liver disease was identified when both liver–kidney contrast (bright liver) and vascular blurring were observed.^17^

### Body composition measurement

Body composition parameters including body fat mass (BFM) and percent body fat (PBF) were measured using a bioimpedance analysis device (InBody S20, Biospace, Seoul, Korea), which appears to be noninvasive and accurate for evaluating body composition.^18^

### Blood examination

Blood samples were collected after overnight fasting and used for the liver and renal function tests and for the measurement of the levels of serum fasting blood glucose (FBG), insulin, hemoglobin A1c and lipids. Biochemical measurements were performed by SRL Inc. (Tokyo, Japan). The insulin resistance index was calculated from the homeostasis model assessment of insulin resistance (HOMA-R) using the following equation: (fasting insulin in μU l^−1^) × (FBG in mmol l^−1^)/22.5.^19^ Atherogenic index was calculated by the logarithm with the base 10 of the ratio of serum triglyceride (TG) to high-density lipoprotein cholesterol (HDL-C) in mmol l^−1^.^20^

### Lipid extraction

Serum (100 μl) was mixed with 1 ml of chloroform, 2 ml of methanol and 0.6 ml of 0.9% potassium chloride (KCl) solution. Then, 1 nmol of C17 SM and C17 Cer (Avanti Polar Lipids, Alabaster, AL, USA) as the internal standard for LC-MS/MS was added. After shaking the solution for 2 h at 37 °C, 1 ml of chloroform and 1 ml of 0.9% KCl solution were added, followed by centrifugation for 5 min at 1600* g*. The lower phase was collected and the residue was re-extracted. After drying under a stream of nitrogen, the resulting lipid film was dissolved in 1.5 ml of a mixture of methanol and chloroform (9:1, v/v). To remove glycerolipids, 150 μl of 4 M potassium hydroxide (KOH) in 70% methanol was added, and the resulting mixture was incubated at 37 °C for 2 h. Then, 1.3 ml of chloroform, 1.36 ml of 0.9% KCl and 50 μl of acetic acid were added to bring the solution to neutral pH. After centrifugation for 5 min at 1600 *g*, the lower phase was collected. Again, 1.5 ml of chloroform was added to the residue phase and the residue was extracted. The lower phase was harvested and dried under a stream of nitrogen. The resulting lipid film was dissolved in 100 μl of acetonitrile and methanol (9:1, v/v).

### Mass spectrometry

We suitably modified the method of Merrill *et al.*^21^ and measured the levels of SM and Cer molecular species, using a TripleTOF 5600 System (AB SCIEX, Foster City, CA, USA) equipped with an electrospray ionization probe and interfaced with a Prominence UFLC system (Shimadzu, Kyoto, Japan). Lipid extracts of 5 μl (equivalent to 5 μl of serum) were injected into a column (InertSustain NH2, 5 μm, 2.1 i.d. × 150 mm, GL Science, Tokyo, Japan). Mobile phase A was acetonitrile/methanol/formic acid (95:5:0.2, v/v) containing 5 mM ammonium formate and mobile phase B was methanol/formic acid (100:0.2, v/v) containing 5 mM ammonium formate. A sample was eluted at 0.2 ml min^−1^ through a 45-min gradient: mobile phase B, 0–5 min 0%, 5–10 min from 0 to 20%, 10–12 min hold 20%, 12–15 min from 20 to 50%, 15–22 min hold 50%, 22–27 min from 50 to 80%, 27–30 min hold, 30–45 min from 80 to 0%. LC-MS/MS was performed in the positive ion mode. Monitoring ions of each of the SM and Cer molecular species were selected for the product ion at *m/z* 184 and 264, respectively. SM and Cer molecular species were quantified as ratios to their C17 internal standard peak areas of product ions. Total SM was calculated from the sum of the levels of d18:1/C14:0, d18:1/C16:0, d18:1/C16:1, d18:1/C18:0, d18:1/C18:1, d18:1/C20:0, d18:1/C20:1, d18:1/C22:0, d18:1/C22:1, d18:1/C24:0 and d18:1/C24:1 SM molecular species. Total Cer was calculated from the sum of the levels of d18:1/C16:0, d18:1/C18:0, d18:1/C20:0, d18:1/C22:0, d18:1/C24:0 and d18:1/C24:1 Cer molecular species.

### Statistical analysis

Statistical analysis was performed using Statistical Analysis version 2.0 (Esumi, Tokyo, Japan). The results were expressed as mean ±s.d. Significant differences between two groups were evaluated by Student's *t-test*. Correlations were analyzed using Spearman's rank correlation. In all the tests, a *P*-value of <0.05 was considered significant.

## Results

### Patient characteristics

The characteristics of the 11 control and 12 obese subjects are shown in Table 1. Both the groups had similar age distributions (*P*=0.181). The BFM and PBF were significantly higher in the obese group than in the control group. A fatty change of the liver was not observed in the control group. However, it was observed in 9 out of 12 subjects in the obese group on ultrasonography—these nine were diagnosed as having NAFLD. Although the levels of FBG and hemoglobin A1c were not significantly different between the groups, the levels of immunoreactive insulin (IRI) and HOMA-R were significantly higher in the obese group. The levels of liver enzymes in serum such as aspartate aminotransferase, alanine aminotransferase, γ-glutamyl transpeptidase (γ-GTP) and cholinesterase (ChE), were significantly higher in the obese group. The low-density lipoprotein cholesterol (LDL-C), TG and uric acid levels were significantly higher in the obese group than in the control group. On the other hand, the amylase and HDL-C levels were significantly higher in the control group than in the obese group. Atherogenic index was significantly higher in obese group. The total protein, albumin, total cholesterol (T-chol), blood urea nitrogen, creatinine and C-reactive protein (CRP) levels were comparable between the groups.

### Analysis of total SM and Cer levels in serum from the control and obese groups

**Figure 1:**
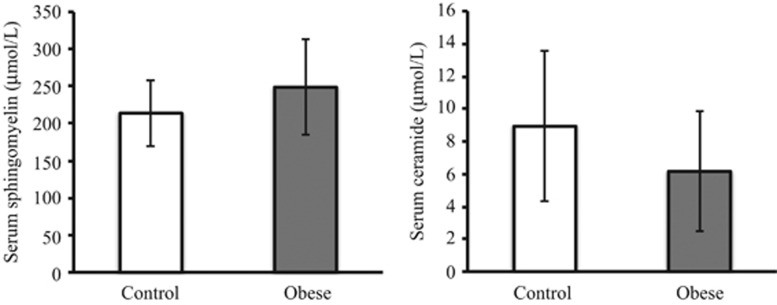
Total serum SM and Cer levels in the control and obese groups. Total SM and Cer levels were measured by LC-MS. The results are expressed as mean±s.d.

To determine the relationship between obesity and sphingolipids, we extracted total sphingolipids from the serum from the obese and control volunteers and measured the total levels of SM and Cer by LC-MS/MS. The obese group tended to have a higher SM level and a lower Cer level; however, these differences were not statistically significant (Figure 1).

### The levels of SM molecular species containing saturated acyl chains are high in the obese group

We next analyzed the molecular species of SM and Cer in the serum from the obese and control volunteers. We detected 11 species of SM (C14:0, C16:0, C16:1, C18:0, C18:1, C20:0, C20:1, C22:0, C22:1, C24:0 and C24:1) and six species of Cer (C16:0, C18:0, C20:0, C22:0, C24:0 and C24:1) in serum obtained from both the groups (Table 2). The levels of SM C14:0 and C16:0 did not differ significantly between the two groups. The levels of SM C18:0 and C24:0 were significantly high in the obese group. Moreover, the levels of SM C20:0 and C22:0 tended to be high in the obese group. Interestingly, the levels of SM species containing unsaturated acyl chains (C16:1, C18:1, C20:1, C22:1 and C24:1) in the obese group were not markedly different from those in the control group. The levels of all Cer species (C16:0, C18:0, C20:0, C22:0, C24:0 and C24:1) did not show any significant difference between the two groups. However, the sum of Cer C18:0, C20:0 and C22:0 tended to be lower in the obese group (*P*=0.095, data not shown).

### The levels of SM molecular species containing saturated acyl chains correlate with parameters of obesity

To investigate in detail the association between serum SM molecular species and obesity, we examined the correlation between the levels of SM molecular species and obesity parameters. SM species with saturated acyl chains (C18:0, C20:0, C22:0 and C24:0) positively correlated with PBF (Table 3). On the other hand, all SM species containing unsaturated acyl chains (C16:1 C18:1, C20:1, C22:1 and C24:1) did not strongly correlate with PBF, BFM or BMI. The levels of SM C18:0, C20:0, C22:0 and C24:0 showed significant correlation with BFM, and the levels of SM C18:0 and C22:0 showed significant correlation with BMI. Moreover, SM C20:0 (*P*=0.052) and C24:0 (*P*=0.055) only tended to correlate with BMI.

Subsequently, we examined the correlation between the levels of Cer species and obesity parameters. Cer C22:0 level negatively correlated with BMI (Table 4). Cer C24:1 level significantly correlated with BFM and PBF. However, Cer C18:0, C20:0 and C24:0 levels did not significantly correlate with BMI, BFM or PBF. In addition, the sum of Cer C18:0, C20:0 and C22:0 did not significantly correlate with these parameters (data not shown). These results suggest that SM species containing the distinct saturated acyl chains are related to the development of obesity.

### SM molecular species with saturated acyl chains are associated with the parameters of insulin resistance

We next examined the correlation between SM species and the parameters of insulin resistance. The levels of SM C14:0 and C16:0 did not correlate with HOMA-R (Table 3); however, the levels of SM C18:0, C20:0, C22:0 and C24:0 significantly correlated with HOMA-R. Moreover, these SM species also correlated with IRI. On the other hand, all SM species containing unsaturated acyl chains (C16:1, C18:1, C20:1, C22:1 and C24:1) did not correlate with HOMA-R or IRI level.

Regarding Cer, all its species measured did not correlate with IRI level or HOMA-R (Table 4). Moreover, the sum of Cer C18:0, C20:0 and C22:0 did not significantly correlate with these parameters (data not shown). These results suggest that SM species with saturated acyl chains are related to the development of insulin resistance.

### SM molecular species with saturated acyl chains are associated with impaired liver function

The development of obesity and insulin resistance is strongly associated with the pathogenesis of fatty liver disease.^22^ Therefore, we next examined whether there is a correlation between SM molecular species and the parameters of liver function. The levels of SM C14:0 and C16:0 did not correlate with any parameters of liver function (Table 3). However, the levels of SM C18:0, C20:0, C22:0 and C24:0 positively correlated with ChE. Moreover, the levels of SM C20:0, C22:0 and C24:0 correlated with aspartate aminotransferase, alanine aminotransferase and γ-GTP levels. On the other hand, SM species containing unsaturated acyl chains (C16:1, C18:1, C20:1, C22:1 and C24:1) showed no significant correlation with the parameters of liver function.

We investigated the correlation between the levels of Cer species and the parameters of liver function. Cer C24:0 and C24:1 levels positively correlated with γ-GTP and ChE, respectively (Table 4). Cer C22:0 levels negatively correlated with ChE. However, Cer C18:0 and C20:0 levels did not clearly correlate with the parameters of liver function. The sum of Cer C18:0, C20:0 and C22:0 did not significantly correlate with these parameters (data not shown). Because 9 out of the 12 obese subjects were diagnosed as having NAFLD, these results suggest an association between the high levels of SM species containing saturated acyl chains and the pathogenesis of NAFLD.

### SM molecular species containing saturated acyl chains correlate with atherogenic dyslipidemia

We examined whether the parameters of lipid metabolism correlate with the levels of SM molecular species in serum. The levels of SM C14:0 and C16:0 did not correlate with T-chol or TG level (Table 3); however, the levels of SM C18:0, C20:0, C22:0 and C24:0 significantly correlated with T-chol and TG levels. Moreover, the levels of these SM species with saturated acyl chains significantly correlated with the LDL-C level. On the other hand, the levels of SM species with unsaturated acyl chains (C:16:1, C18:1, C20:1, C22:1 and C24:1) were not associated with T-chol, TG or LDL-C level. Although we examined the correlation between SM species and HDL-C level, none of the SM species significantly correlated with HDL-C level (Table 3). Onat *et al.*^23^ indicated that the atherogenic index (log [TG/HDL-C]) is an independent predictive value for cardiovascular disease. Thus, we finally examined whether the atherogenic index correlates with the levels of SM molecular species in serum. The levels of SM C14:0 and C16:0 did not correlate with atherogenic index (Table 3). However, the levels of SM C18:0, C20:0, C22:0 and C24:0 significantly correlated with the atherogenic index.

Cer C22:0 level correlated with LDL-C and HDL-C levels, and Cer C24:1 level was associated with atherogenic index, TG and HDL-C levels (Table 4). However, Cer C18:0 and C20:0 levels did not correlate with lipid metabolism parameters and atherogenic index. In addition, the sum of Cer C18:0, C20:0 and C22:0 did not significantly correlate with these parameters (data not shown). These results suggest that the increase in the levels of SM species with saturated acyl chains is involved in the pathogenesis of atherogenic dyslipidemia.

## Discussion

In this study, we observed that the levels of SM species with saturated acyl chains (C18:0, C20:0, C22:0 and C24:0) in serum were high in young obese adults, and most of these SM species positively correlated with the parameters of obesity, insulin resistance, liver function and atherogenic dyslipidemia. These results suggest that the increase in the levels of SM species with distinct saturated acyl chains is related to the development of obesity-related diseases such as insulin resistance, NAFLD and atherogenic dyslipidemia.

A number of previous studies have demonstrated the composition of SM and Cer species in human serum and plasma. Hammad *et al.*^24^ showed that SM C16:0 and C24:1 are the predominant SM species in healthy human serum. In this study, the predominant SM species in both the control and obese groups were also C16:0 and C24:1 (Table 2). Therefore, these predominant SM species may not be affected by the development of obesity. On the other hand, the levels of SM species containing saturated acyl chains (C18:0, C20:0, C22:0 and C24:0) were high in the obese group and many of these SM species significantly correlated with the obesity parameters, namely, BMI, PBF and BFM (Tables 2 and 3). However, the total SM levels were not significantly different between the control and obese groups (Figure 1). Thus, we consider that the changes in SM molecular composition rather than quantitative variations in SM are associated with the development of obesity. On the other hand, the major Cer species in the both control and obese groups were C22:0 and C24:1. Previous studies showed that dominant Cer species were C24:0 and C24:1 in human serum and plasma.^15,24^ In the SM and Cer species measurements, we suitably modified the method of Merrill *et al.*^21^ and analyzed SM and Cer species by scheduled multiple reaction monitoring, which is a tool for increasing the efficiency and accuracy of quantitative MS/MS. The reason why our results are inconsistent with previous reports may be the difference in sample preparation, analysis methods or the clinical profiles of the subjects.

Previous studies showed the relationship between obesity and sphingolipid metabolism.^11,12^ In addition, obesity is associated with a chronic low-grade inflammation.^25^ Tumor necrosis factor-α, an inflammatory cytokine, is released from macrophages and adipocytes, and is involved in the increase in Cer level by activating sphingomyelinase.^26^ In the present study, the total Cer level in the obese group did not increase (Figure 1) and the levels of almost all Cer species did not correlate with obesity parameters (Table 4). This may be due to the absence of severe inflammation in young obese adults. Indeed, although we did not measure high-sensitivity CRP level, the level of CRP in the obese group was not significantly higher than that in the control group (Table 1). However, the levels of Cer C18:0 and C20:0 species tended to be negatively correlated with BMI, γ-GTP and ChE, whereas the levels of C24:0 and C24:1 tended to be positively correlated with these parameters, although not statistically significant (Table 4). These parallel correlation changes indicate that the proinflammatory state and autoimmune activation may be mediated by sphingolipid metabolism.

We demonstrated that the levels of SM species containing saturated acyl chains (C18:0, C20:0, C22:0 and C24:0) positively correlated with the parameters of insulin resistance (Table 3). Onat *et al.*^27^ reported that plasma polypeptides and proteins suffered from damage in their epitopes aggregated to protective plasma protein in autoimmune activation state, and partially leaked from quantitative assay. In this study, Cer C18:0 to C22:0 species tended to be lower in the obese group (*P*=0.095, data not shown), suggesting that these Cer species may be aggregated to protective plasma protein by autoimmune activation. For this reason, the Cer species that we measured may not show any correlation with IRI level or HOMA-R (Table 4). Haus *et al.*^16^ reported the relationship between plasma Cer and insulin resistance in patients with type 2 diabetes. They also indicated that insulin sensitivity inversely correlates with Cer C18:0, C20:0, C24:0 and C24:1 levels. Moreover, Brozinick *et al.*^28^ demonstrated that the total Cer level and the levels of the Cer species C14:0, C16:0, C22:0 and C24:0 are high in prediabetic and diabetic nonhuman primates, and that the levels of these Cer species correlate with HOMA-R. In this study, although IRI level and HOMA-R in the obese group were higher than those in the control group (Table 1), the obese participants had not yet developed diabetes. The tumor necrosis factor-α level has been shown to be high in type 2 diabetes.^29^ Therefore, it is interesting to speculate that these young obese adults will develop diabetes in the future, and sphingomyelinase is activated by tumor necrosis factor-α and hydrolyzes SM species containing saturated acyl chains. Then, the levels of Cer species containing saturated acyl chains might increase and correlate with insulin resistance parameters.

The progression of insulin resistance contributes to the pathogenesis of NAFLD.^10^ In our results, many of the SM species containing saturated acyl chains positively correlated with the parameters of liver function (aspartate aminotransferase, alanine aminotransferase, γ-GTP and ChE levels, Table 3), suggesting that the increase in the levels of these SM species is related to the pathogenesis of NAFLD. Li *et al.*^30^ indicated that the elevation of the level of SM, which is induced by liver-specific SM synthase 2 overexpression, promotes fatty acid uptake and liver steatosis in mice. On the other hand, Pagadala *et al.*^31^ demonstrated that the increase in the level of Cer is associated with the pathogenesis of NAFLD and the progression to NASH. Therefore, further studies are needed to elucidate the relationship between sphingolipid metabolism and NAFLD.

The parameters of lipid metabolism such as cholesterol, TG and LDL-C levels are high in atherogenic dyslipidemia. Previous studies demonstrated that total SM level in blood positively correlates with the levels of TG and LDL-C.^32,33^ However, the relationship between SM/Cer species and lipid metabolism parameters was not well defined. In the present study, Cer C22:0 level negatively correlated with LDL-C levels (Table 4). Moreover, yet without reaching significance, Cer C18:0 and C20:0 species showed the negative correlation with cholesterol and LDL-C levels. On the other hand, there was a tendency to positive correlation between the levels of Cer C24:0 and C24:1 species and cholesterol and LDL-C levels, although statistically insignificant. These results suggest that the inverse relationship between lipoprotein levels and Cer C18:0, C20:0 and C22:0 species levels may exist. In addition, we showed that the levels of SM species containing saturated acyl chains correlate with atherogenic index, cholesterol, TG and LDL-C levels (Table 3), suggesting that these SM species are related to the development of atherogenic dyslipidemia. Previous studies demonstrated that high SM level is a risk factor for coronary artery disease.^14^ Thus, high levels of SM species containing saturated acyl chains, but not high total SM level, may be a risk factor for coronary artery disease.

SM is generated from Cer and phosphatidylcholine by SM synthase activity, raising the possibility that not only alteration of sphingolipids, but also alteration of phospholipids may be related to the pathogenesis of metabolic syndrome. Indeed, Onat *et al.*^34^ indicated that excessive total phospholipids may mediate inflammation, and excessive HDL phospholipids are independent predictors of the risk of metabolic syndrome in each gender. Thus, it is also an important matter to analyse a class and molecular species of phospholipid in the obese group.

Although the detailed mechanism by which the levels of these SM species increase in the obese group is unclear, a challenging subject of future studies is determining in the obese groups the activities of enzymes that modulate sphingolipid molecular species, such as SM metabolic enzymes (SMS and sphingomyelinase), Cer synthases, Elovl family (elongation of very long chain fatty acids) enzymes and fatty acid desaturases.

In conclusion, the levels of SM species containing saturated acyl chains (C18:0, C20:0, C22:0 and C24:0) are high in young obese adults. Moreover, many of these SM species are associated with obesity-related diseases such as insulin resistance, NAFLD and atherogenic dyslipidemia. Therefore, these SM species could serve as novel biomarkers of these obesity-related diseases.

## Figures and Tables

**Table 1:** Clinical characteristics of the study subjects

	*Control group (*n*=11)*	*Obese group (*n*=12)*
Gender (M:F)	7:4	12:0
Fatty liver (*n*)	0	9
Age (years)	23.2±2.4	21.7±2.8
BMI (kg m^−^^2^)	19.8±1.1	36.8±4.4**
BFM (kg)	9.1±2.7	42.9±11.3**
PBF (%)	16.4±5.6	39.0±6.0**
FBG (mg dl^−1^)	83.8±4.8	85.3±5.9
HbA1c (%)	5.1±0.4	5.1±0.3
IRI (μU ml^−1^)	3.5±1.2	14.6±8.3**
HOMA-R	0.7±0.3	3.0±1.7**
TP (g dl^−1^)	7.3±0.3	7.4±0.3
Alb (g dl^−1^)	4.7±0.2	4.5±0.2
T-bil (mg dl^−1^)	1.0±0.4	1.0±0.5
AST (U l^−1^)	18.6±6.1	31.8±20.3*
ALT (U l^−1^)	17.3±10.8	57.5±50.4*
γGTP (IU l^−1^)	18.0±4.9	44.5±32.9*
ChE (U l^−1^)	275.0±47	407.3±55.0**
Amy (U l^−1^)	89.6±48.4	56.8±12.2
T-chol (mg dl^−1^)	171.6±19.9	204.8±71.4
HDL-C (mg dl^−1^)	64.6±13.6	40.3±5.2**
LDL-C (mg dl^−1^)	90.4±24.5	143.3±60.4*
TG (mg dl^−1^)	62.6±38.3	130.1±75.5*
Atherogenic index	0.30±0.26	0.81±0.28**
UA (mg dl^−1^)	5.0±1.7	7.2±1.3**
BUN (mg dl^−1^)	12.2±1.9	11.0±2.4
Cre (mg dl^−1^)	0.8±0.1	0.8±0.1
CRP (mg dl^−1^)	0.1±0.1	0.5±0.8

Abbreviations: Alb, albumin; ALT, alanine aminotransferase; Amy, amylase; AST, aspartate aminotransferase; BFM, body fat mass; BMI, body mass index; BUN, blood urea nitrogen; ChE, cholinesterase; Cre, creatinine; CRP, C-reactive protein; F, female; FBG, fasting blood glucose; HbA1c, hemoglobin A1c; HDL-C, high-density lipoprotein cholesterol; HOMA-R, homeostasis model assessment of insulin resistance; IRI, immunoreactive insulin; LDL-C, low-density lipoprotein cholesterol; M, male; PBF, percentage body fat; T-bil, total bilirubin; T-chol, total cholesterol; TG, triglyceride; TP, total protein; UA, uric acid; γGTP, γ-glutamytranspeptidase.

Atherogenic index was caluculated by the the logarithm to the base 10 of the ratio of serum triglyceride to HDL-C in mmol l^−1^. Values are given as mean ±s.d. The significance of differences between the control and obese groups was analyzed by Student's *t*-test. *0.01<*P*<0.05, **P<0.01.

**Table 2:** Serum sphingomyelin and ceramide molecular species in control and obese groups

*Sphingomyelin molecular species*	*Control group sphingomyelin (μmol l^−1^)*	*Obese group sphingomyelin (μmol l^−1^)*	P-*value*
C14:0	12.9±4.9	12.9±5.4	0.994
C16:0	37.9±8.5	44.1±11.7	0.163
C16:1	25.4±7.4	30.5±11.1	0.208
C18:0	12.4±2.0	15.6±3.0	**0.008**
C18:1	19.6±7.0	23.9±8.6	0.201
C20:0	7.6±1.5	9.1±2.3	0.084
C20:1	11.2±4.6	12.0±4.3	0.680
C22:0	12.5±2.1	14.9±3.6	0.060
C22:1	26.7±7.0	29.6±9.0	0.392
C24:0	11.4±1.8	14.4±3.2	**0.012**
C24:1	36.2±7.2	41.2±11.7	0.187

*Ceramide molecular species*	*Control group ceramide (μmol l^−1^)*	*Obese group ceramide (μmol l^−1^)*	P*-value*
C16:0	0.39±0.09	0.46±0.12	0.136
C18:0	0.37±0.25	0.26±0.13	0.199
C20:0	0.718±0.51	0.50±0.38	0.275
C22:0	5.28±3.88	2.36±2.94	0.058
C24:0	0.763±0.16	0.83±0.27	0.453
C24:1	1.41±0.46	1.74±0.38	0.073

Values are given as mean ±s.d. The significance of differences between the control and obese groups was analyzed by Student's *t*-test. Bold indicates a statistically significant difference between the control and obese groups.

**Table 3:** Spearman's rank correlations between sphingomyelin molecular species and various parameters

	*SM C14:0*		*SM C16:0*		*SM C18:0*		*SM C20:0*		*SM C22:0*		*SM C24:0*	
	r	P*-value*	r	P*-value*	r	P*-value*	r	P*-value*	r	P*-value*	r	P*-value*
BMI (kg m^−^^2^)	−0.013	0.952	0.177	0.419	0.493	**0.017**	0.410	0.052	0.417	**0.048**	0.406	0.055
BFM (kg)	0.204	0.351	0.092	0.677	0.573	**0.004**	0.423	**0.045**	0.485	**0.019**	0.449	**0.032**
PBF (%)	0.297	0.169	0.166	0.449	0.661	**0.001**	0.451	**0.031**	0.521	**0.011**	0.525	**0.010**
IRI (μU ml^−1^)	0.145	0.510	0.178	0.415	0.497	**0.016**	0.457	**0.028**	0.451	**0.031**	0.514	**0.012**
HOMA-R	0.171	0.435	0.221	0.310	0.525	**0.010**	0.461	**0.027**	0.482	**0.020**	0.552	**0.006**
AST (U l^−1^)	−0.126	0.567	0.222	0.309	0.377	0.076	0.427	**0.042**	0.453	**0.030**	0.589	**0.003**
ALT (U l^−1^)	−0.145	0.508	0.280	0.196	0.389	0.067	0.447	**0.033**	0.489	**0.018**	0.625	**0.001**
γGTP (IU l^−1^)	−0.089	0.688	0.309	0.152	0.382	0.072	0.491	**0.017**	0.528	**0.010**	0.613	**0.002**
ChE (U l^−1^)	0.049	0.825	0.154	0.484	0.497	**0.016**	0.452	**0.030**	0.435	**0.038**	0.575	**0.004**
T-chol (mg dl^−1^)	0.126	0.566	0.243	0.264	0.551	**0.006**	0.642	**0.001**	0.618	**0.002**	0.678	**0.0004**
HDL-C (mg dl^−1^)	−0.005	0.982	−0.191	0.383	−0.369	0.083	−0.320	0.137	−0.321	0.136	−0.357	0.094
LDL-C (mg dl^−1^)	−0.015	0.945	0.300	0.164	0.477	**0.021**	0.553	**0.006**	0.498	**0.016**	0.613	**0.002**
TG (mg dl^−1^)	0.371	0.082	−0.024	0.913	0.564	**0.005**	0.581	**0.004**	0.559	**0.006**	0.476	**0.022**
Atherogenic index	0.275	0.205	0.048	0.826	0.523	**0.010**	0.517	**0.012**	0.501	**0.015**	0.459	**0.027**

	*SM C16:1*		*SM C18:1*		*SM C20:1*		*SM C22:1*		*SM C24:1*		*Total*	
	r	P*-value*	r	P*-value*	r	P*-value*	r	P*-value*	r	P*-value*	r	P*-value*
BMI (kg m^−^^2^)	0.091	0.678	0.066	0.764	−0.047	0.832	0.058	0.793	0.114	0.604	0.260	0.230
BFM (kg)	0.244	0.263	0.241	0.268	0.126	0.568	0.150	0.494	0.135	0.539	0.338	0.115
PBF (%)	0.305	0.157	0.343	0.109	0.226	0.299	0.245	0.260	0.220	0.313	0.427	**0.042**
IRI (μU ml^−1^)	0.284	0.189	0.219	0.315	0.128	0.562	0.136	0.535	0.210	0.337	0.360	0.092
HOMA-R	0.274	0.206	0.178	0.417	0.073	0.740	0.130	0.553	0.226	0.299	0.363	0.089
AST (U l^−1^)	−0.083	0.706	−0.100	0.651	−0.145	0.510	−0.032	0.886	0.133	0.546	0.147	0.503
ALT (U l^−1^)	−0.021	0.923	−0.091	0.680	−0.169	0.442	0.036	0.870	0.205	0.347	0.229	0.293
γGTP (IU l^−1^)	−0.091	0.680	−0.203	0.352	−0.265	0.222	−0.010	0.962	0.184	0.401	0.222	0.309
ChE (U l^−1^)	0.147	0.504	0.085	0.701	−0.009	0.968	0.045	0.839	0.142	0.519	0.260	0.231
T-chol (mg dl^−1^)	−0.007	0.973	0.013	0.952	0.026	0.906	0.068	0.757	0.109	0.620	0.265	0.222
HDL-C (mg dl^−1^)	−0.222	0.308	−0.163	0.458	−0.032	0.884	−0.101	0.647	−0.152	0.488	−0.243	0.264
LDL-C (mg dl^−1^)	−0.002	0.993	−0.033	0.881	−0.116	0.599	0.026	0.906	0.122	0.579	0.245	0.261
TG (mg dl^−1^)	0.311	0.149	0.330	0.124	0.263	0.225	0.153	0.485	0.086	0.698	0.317	0.141
Atherogenic index	0.293	0.174	0.287	0.185	0.185	0.399	0.149	0.497	0.111	0.615	0.311	0.148

Abbreviations: ALT, alanine aminotransferase; AST, aspartate aminotransferase; BFM, body fat mass; BMI, body mass index; ChE, cholinesterase; HDL-C, high-density lipoprotein cholesterol; HOMA-R, homeostasis model assessment of insulin resistance; IRI, immunoreactive insulin; LDL-C, low-density lipoprotein cholesterol; PBF, percentage body fat; T-chol, total cholesterol; TG, triglyceride; γGTP, γ-glutamytranspeptidase.

Bold indicates that the correlation is statistically significant. Atherogenic index was calculated by the logarithm to the base 10 of the ratio of serum triglyceride to HDL-C in mmoll^−1^.

**Table 4:** Spearman's rank correlations between ceramide molecular species and various parameters

	*Cer C16:0*		*Cer C18:0*		*Cer C20:0*		*Cer C22:0*		*Cer C24:0*		*Cer C24:1*		*Total*	
	r	P*-value*	r	P*-value*	r	P*-value*	r	P*-value*	r	P*-value*	r	P*-value*	r	P*-value*
BMI (kg m^−^^2^)	0.320	0.136	−0.158	0.471	−0.180	0.411	−0.444	**0.034**	0.177	0.418	0.372	0.080	−0.340	0.112
BFM (kg)	0.343	0.109	0.002	0.991	0.002	0.991	−0.272	0.209	0.299	0.166	0.448	**0.032**	−0.096	0.662
PBF (%)	0.412	0.051	0.053	0.809	0.046	0.833	−0.263	0.226	0.213	0.328	0.459	**0.028**	−0.075	0.733
IRI (μU ml^−1^)	0.225	0.302	−0.203	0.354	−0.184	0.400	−0.333	0.120	0.321	0.136	0.343	0.109	−0.236	0.278
HOMA-R	0.235	0.280	−0.201	0.359	−0.201	0.359	−0.338	0.115	0.363	0.089	0.395	0.062	−0.223	0.306
AST (U l^−1^)	0.036	0.870	−0.355	0.097	−0.405	0.055	−0.338	0.115	0.382	0.072	0.194	0.376	−0.300	0.164
ALT (U l^−1^)	0.168	0.443	−0.263	0.226	−0.333	0.121	−0.339	0.114	0.359	0.092	0.297	0.169	−0.288	0.183
γGTP (IU l^−1^)	0.203	0.353	−0.216	0.322	−0.236	0.277	−0.275	0.204	0.431	**0.040**	0.288	0.183	−0.223	0.307
ChE (U l^−1^)	0.323	0.133	−0.200	0.360	−0.192	0.380	−0.452	**0.030**	0.216	0.322	0.450	**0.031**	−0.301	0.163
T-chol (mg dl^−1^)	0.320	0.136	−0.214	0.328	−0.102	0.642	−0.259	0.233	0.196	0.371	0.118	0.591	−0.133	0.545
HDL-C (mg dl^−1^)	−0.212	0.333	0.270	0.213	0.265	0.221	0.464	**0.026**	−0.154	0.482	−0.429	**0.041**	0.351	0.101
LDL-C (mg dl^−1^)	0.361	0.090	−0.353	0.099	−0.256	0.237	−0.440	**0.036**	0.149	0.497	0.295	0.172	−0.314	0.145
TG (mg dl^−1^)	0.221	0.312	−0.211	0.334	−0.180	0.411	−0.375	0.078	0.342	0.111	0.456	**0.029**	−0.266	0.220
Atherogenic index	0.271	0.211	−0.193	0.378	−0.178	0.417	−0.411	0.051	0.254	0.242	0.512	**0.013**	−0.286	0.187

Abbreviations: ALT, alanine aminotransferase; AST, aspartate aminotransferase; BFM, body fat mass; BMI, body mass index; ChE, cholinesterase; HDL-C, high-density lipoprotein cholesterol; HOMA-R, homeostasis model assessment of insulin resistance; IRI, immunoreactive insulin; LDL-C, low-density lipoprotein cholesterol; PBF, percentage body fat; T-chol, total cholesterol; TG, triglyceride; γGTP, γ-glutamytranspeptidase.

Bold indicates that the correlation is statistically significant. Atherogenic index was calculated by the logarithm to the base 10 of the ratio of serum triglyceride to HDL-C in mmol l^−1^.
